# Level of Potassium Is Associated with Saturated Fatty Acids in Cell Membranes and Influences the Activation of the 9 and 13 HODE and 5 HETE Synthesis Pathways in PCOS

**DOI:** 10.3390/biomedicines10092244

**Published:** 2022-09-09

**Authors:** Małgorzata Szczuko, Kamila Pokorska-Niewiada, Lidia Kwiatkowska, Jolanta Nawrocka-Rutkowska, Iwona Szydłowska, Maciej Ziętek

**Affiliations:** 1 Department of Human Nutrition and Metabolomics, Pomeranian Medical University in Szczecin, 71-460 Szczein, Poland; 2 Department of Toxicology, Dairy Technology and Food Storage, West Pomeranian University of Technology in Szczecin, 71-374 Szczecin, Poland; 3 Department of Gynecology, Endocrinology and Gynecological Oncology, Pomeranian Medical University Szczecin, 71-252 Szczecin, Poland; 4 Department of Perinatology, Obstetrics and Gynecology, Pomeranian Medical University in Szczecin, 72-009 Police, Poland

**Keywords:** erythrocytes, potassium, HODE, HETE, arachidonic acid derivatives (AA)

## Abstract

Potassium helps to maintain the water–electrolyte and acid–base balance. There is little research on the relationship between plasma fatty acids (FAs), inflammatory mediators and red blood cell potassium levels in women with polycystic ovary syndrome (PCOS). This study included 38 Caucasian women with PCOS. Potassium in the erythrocytes was determined by inductively coupled atomic plasma emission spectrometry. The FAs were analysed with gas chromatography, and liquid chromatography was used to separate the eicosanoids. The relationships between the potassium content and the amounts of fatty acids, as well as potassium and arachidonic acid (AAs) derivatives, were analysed. Significant negative correlations were found with, among others, pentadecanoic acid, palmitic acid, stearic acid and arachidic acid, whereas a positive correlation was found with neuronic acid. Positive correlations were observed with 9, 13 HODE (derivatives synthetized from linolenic acid) and 5 oxo ETE and 5 HETE (from 5 LOX pathway). Saturated fatty acids reduce the influx of potassium into the cell by destabilizing the pH of the cytosol, and thus exacerbating the inflammatory response through the activation of the AA cascade. Therefore, improving the flow of potassium inside the cell is important in the treatment of patients.

## 1. Introduction

Polycystic ovary syndrome (PCOS) is a common endocrine disease that affects over 10% of women of childbearing age [1]. Metabolic abnormalities caused by PCOS, such as increased abdominal fat and insulin resistance, contribute to further complications, such as cardiovascular disease, heart attack, stroke and type 2 diabetes [2].

It has been proven that many diseases, including infertility, are associated with the incorrect metabolism of elements [3]. An element of crucial importance in the water and electrolyte balance is potassium, which is one of the most important components of the protoplasm of cells. It is present in small amounts in body fluids (plasma, lymph) [4]. The analysis of the level of this element in erythrocytes is the best single measure of the state of this element in the body [5]. Potassium is an element that increases the permeability of the cell membrane (it is a calcium antagonist), increases the muscle tone and activity of the secretory glands and plays a decisive role in maintaining the excitability of nerve tissues. The distribution of potassium between the intracellular and extracellular fluid compartments is regulated by physiological factors, such as insulin and catecholamines, which stimulate the activity of Na^+^/K^+^ ATPase [6]. Potassium and other ions are transported through the lipid bilayer via membrane proteins in one of two methods, without the use of energy (passive transport) or with the use of energy (active transport). K^+^/Cl^−^ and Na^+^/K^+^/2Cl^–^ cotransporters, Na^+^/H^+^ and Na^+^/Mg^2+^ ion exchangers and K^+^ channels activated by increasing intracellular Ca^2+^ concentration (Gardos channels) are responsible for the passive ion flow (gradient). The passive outflow of potassium (5–10%) and the influx of sodium in the red blood cells are balanced by the sodium–potassium pump (Na^+^/K^+^ ATPase), in which, as a result of active transport, two potassium ions are transported into the cell and three sodium ions are removed from it [7]. With large fluctuations in the concentration of cations, the volume of blood cells changes. When the sodium leakage exceeds the potassium leakage, the erythrocytes swell and contract when the outward potassium leakage exceeds the sodium inflow. Moreover, an increase in the amount of cations increases the inflow of water to the blood cells and causes an increase in their volume, and a decrease causes the dehydration and shrinkage of blood cells [8,9]. Changes in erythrocyte hydration affect their shape and deformation capacity, which in turn allows the erythrocytes to carry oxygen. In addition to carrying oxygen and carbon dioxide, erythrocytes also have immune functions, such as enhancing phagocytosis, defending against infection, increasing immune adhesion, recognizing and carrying antigens and removing circulating immune complexes [10]. 

Red blood cells are responsible for the oxidative damage caused by hydrogen peroxide (H_2_O_2),_ due to their role in oxygen (O_2_) transport. In red blood cells, H_2_O_2_ causes both lipid peroxidation and iron oxidation in haemoglobin to form methaemoglobin. A study of lipid peroxidation showed an increase in the fragility of the cell membrane, which may contribute to changes in potassium transport or be the result of changes in the transport of this element [11].

Arachidonic acid (AA) and linolenic acid (LA), belonging to the omega 6 family of polyunsaturated fatty acids (PUFA), are among the main mediators of inflammation. LA is classified as an exogenous fatty acid (it is an essential component of the diet) and is a precursor of AA. Arachidonic acid (AA) is a key fatty acid component of phospholipids in cell membranes and is also used as a precursor for the synthesis of eicosanoids. These biologically important compounds (prostanoids and leukotrienes) are involved in many physiological and pathophysiological processes, including inflammation. Inflammatory processes are critical to human survival and occur in response to stimuli such as injury or infection [12,13].

There is limited information on the relationships between plasma fatty acids (FAs), inflammatory mediators and red blood cell potassium levels in women with PCOS. The aim of this study was to assess the degree of dependence between the concentration of potassium in erythrocytes and the levels of unsaturated fatty acids in women with PCOS. This work is one of the first studies to detail the relationships between trace elements and levels of inflammatory mediators, but the process has not been fully elucidated. Our observations contribute to the understanding of the processes that take place in the bodies of women with PCOS, and thus have an impact on the course of research on the efforts to fight this disease.

## 2. Materials and Methods

### 2.1. Study Group

This study included 38 Caucasian women of reproductive age (18–38 years), diagnosed with polycystic ovary syndrome using ultrasound (Ultrasound Voluson 730, GE, Basel, Switzerland), according to the Rotterdam criteria [14]. The characteristics of the studied women are shown in Table 1. The research was carried out at the Gynecology and Urogynecology Clinic of the Pomeranian Medical University after obtaining the consent of the Bioethics Committee of the Pomeranian Medical University in Szczecin (No. KB-0012/134/12, with the appendix to the permit No. KB-0012/36/14). All respondents gave their written informed consent, and their confidentiality and anonymity were protected.

The control group (CG) consisted of 17 healthy women with a normal menstrual cycle, with a mean age of 19 to 36 years, who were not hospitalized, had no chronic disease and were not taking any medications, including contraceptives.

### 2.2. Anthropometric Measurements

Height was measured with a portable height meter (with an accuracy of 1 cm). Body weight was measured with an electronic scale (with an accuracy of 0.1 kg). Body mass index (BMI) was calculated using the following formula: the ratio of body weight in kilograms (kg) to the square of height in meters (m^2^). The waist-to-hip ratio (WHR) was calculated by measuring the waist circumference (the narrowest point between the last rib and the pelvic crest at the end of exhalation) and the hip circumference at the widest point, using an anthropometric measuring tape (with an accuracy of 0.5 cm). Body composition and the Na^+^/K^+^ ratio were measured with bioelectrical impedance analysis (BIA-101, Akern, Florence, Italy).

### 2.3. Biochemical Analyses

Biochemical parameters, such as testosterone, insulin and sex hormone-binding globulin (SHBG), were analysed by ECLIA (electrochemiluminescence) and androstenedione by ELISA (Kobas Rosch E411). Biochemical analyses were performed in the university hospital’s laboratory. An amount of 5 mL of blood was taken for analysis; this was then transferred to tubes with ethylenediaminetetraacetic acid (EDTA) and immediately put in the refrigerator. To isolate the morphotic elements, samples were centrifuged at 3500 rpm for 10 min at 4 °C 15 min after collection. Tubes were safe-lock-sealed to prevent the accidental opening of the tube and the loss of the sample (Eppendorf, Germany). The red blood cell samples, as well as plasma samples, were immediately frozen at −80 °C and stored until analysis.

### 2.4. Inductively Coupled Atomic Plasma-Emission Spectrometry (ICP-AES Analysis)

All reagents used for the ICP-AES analyses were of analytical purity. A working standard solution of potassium (Merck KGaA, Darmstadt, Germany) was used for the validation of ICP-AES. In order to ensure the highest quality of the analyses, all vessels were rinsed with 15% HNO_3_ and double-distilled water (Barnstead™ GenPure™ Pro, Thermo Scientific Easypure UV, Hennigsdorf, Germany) and dried before use. For the determination of potassium in the erythrocytes, 1 ± 0.1 mL samples were mineralized in the MDS-2000 microwave oven using 5 mL of nitric acid (69% Merck KGaA, Darmstadt, Germany) and 2 ± 0.1 mL of H_2_O_2_ (35% *w*/*v*). The blanks and the samples with the reference material were mineralized in parallel using the same procedure. The following operating parameters of the furnace were used: maximum power, 1000 W; maximum pressure, 13.8 bar; maximum operating temperature, 200 °C. After the mineralization was completed, the samples were stored in LDPE bottles (Hünersdorff GmbH, Ludwigsburg, Germany) until analysis at 4 °C.

The potassium content in the erythrocytes was determined using the ICP-AES apparatus (Yobin Yvon JY-24 with the Meinhard TR 50-C1 nebulizer, Longjumeau, France). The generator parameters were as follows: output power, 1000 W; frequency, 40.68 MHz; and plasma gas, auxiliary gas and nebulizer gas argon with flow rates of 12.0, 1.0 and 1.1 mL/min, respectively. Potassium was determined at a wavelength of 766.5 nm. All samples were analysed in three analytical duplicates. The accuracy and precision of the methods used were verified with the certified reference material trace elements whole blood L-1 (Seronorm SERO AS, Billingstad, Norway). The limit of quantification (LOQ) was determined on ultrapure blank reagents based on the usual LOQ criteria of 10 blind standard deviations [15]. The LOD for the potassium was 3.55 µg/g, and the LOQ was 13.05 µg/g.

### 2.5. HPLC Analysis

All the chemicals used for the isolation and chromatography analysis (methanol, acetonitrile, HCL, ethyl acetate and acetic acid) were HPLC grade (Sigma-Aldrich, St Louis, MO, USA, and Merck KGaA, Darmstadt, Germany). Double-distilled water was obtained from the MilliQ Water System (Millipore, Billerica, MA, USA). The standards of the analysed arachidonic acid (AAD: 5(S)-HETE (5S-hydroxy-6E,8Z,11Z,14Z-eicosatetraenoic acid), 5(S)-oxoETE (5-oxo-6E,8Z,11Z,14Z-eicosatetraenoic acid), 12(S)-HETE (12S-hydroxy-5Z,8Z,10E, 14Z-eicosatetraenoic acid), 15(S)-HETE (15S-hydroxy-5Z,8Z,11Z,13E-eicosatetraenoic acid), 16(R)-HETE (16R-hydroxy-5Z,8Z,11Z,14Z-eicosatetraenoic acid), 16(S)-HETE (16S-hydroxy-5Z,8Z,11Z,14Z-eicosatetraenoic acid), 5(S),6(R)-lipoxin-A4 (5S,6R,15S-trihydroxy-7E,9E,11Z,13E-eicosatetraenoic acid), 5(S),6(R)15(R)-lipoxin-A4 (5(S),6(R), 15(R)-trihydroxy-7E,9E,11Z,13E-eicosatetraenoic acid), linoleic acid (LAD: 9(S)-HODE (9S-hydroxy-10E,12Z-octadecadienoic acid) and 13(S)-HODE (13S-hydroxy-9Z,11E-octadecadienoic acid) derivatives and internal standard prostaglandin B_2_ (PGB_2_) were obtained from Cayman Chemicals (Ann Arbor, MI, USA). Eicosanoids were extracted from 0.5 mL of plasma, using solid-phase extraction RP-18 SPE columns (Agilent Technologies, Cheadle, UK) [16]. The HPLC separations were performed using an Agilent Technologies 1260 liquid chromatograph. An autosampler (G1329B) was used for the sample injections. The Agilent ChemStation software (Agilent Technologies) was used for the instrument control and the data collection and analysis. The content of buffer B in the mobile phase was 30% at the beginning of the separation, and then it increased to 80% after 20 min of separation and to 98% between 20.1 and 23.9 min; then, it decreased to 30% between 24 and 28 min [17]. The absorbance of the selected compounds was measured at the following wavelengths: 210 nm for 16(R)-HETE and 16(S)-HETE (the latter two were eluted as one peak); 235 nm for 9(S)-HODE, 13(S)-HODE, 5(S)-HETE, 12(S)-HETE and 15(S)-HETE; 280 nm for 5(S)-oxoETE and internal standard prostaglandin B2; and 302 nm for 5(S), 6(R)-lipoxin A4, 5(S), 6 (R) and 15(R)-lipoxin A4. A detailed methodology of detecting eicosanoids is described elsewhere [17].

### 2.6. GC Analysis

The FAs were analysed with the use of gas chromatography (Agilent Technologies 7890A GC System, equipped with a SUPELCOWAX 10 Capillary GC Column). The total analysis time was 16.158 min, and the gas flow rate was 1 mL/min, with nitrogen as the carrier gas. FAs were identified by comparing their retention times with those of the Food Industry FAME Mix (cat. no 35077) (Restek), supplemented with the standards. A detailed methodology of fatty acid detection is described elsewhere [17].

### 2.7. Statistical Analysis

The obtained results were statistically analysed using the Statistica 13.3 software package (Polish version: Statsoft, Kraków, Poland). Data are expressed as the mean with standard deviation (mean ± SD). The profiles of the fatty acids and pro-inflammatory factors were compared with the potassium contents in the erythrocytes of women with PCOS. Pearson’s correlation statistics were used to test the correlations between the variables. To make sure that the performed analyses were correct, Tukey’s post hoc test for the justified significant difference (RIR) was performed, which confirmed the correctness of the analyses. The values above 0.35 and below −0.35 with a *p*-value = 0.05 were calculated as significant for fatty acids and derivatives in Table 2and Table 3.

## 3. Results

By comparing the examined parameters of women with PCOS with the control group, it was found that, despite significant differences in many anthropogenic parameters, the levels of potassium in red blood cells did not differ significantly (Table 1). Then, the correlations of potassium levels in blood cells with the biochemical parameters of women with PCOS (Table 4) were determined, but still no significant relationships were found.

The next stage of our analysis was to check the correlations and possible connections with fatty acids (Table 2). We noticed that there were significant negative correlations with the levels of several saturated fatty acids, including pentadecanoic acid, palmitic acid, stearic acid, arachidic acid cis-11-eicosatrienoic acid and arachidonic acid. However, a positive correlation was found with nervonic acid (Table 2). 

It was shown that the level of potassium in red blood cells positively correlated with derivatives 9, 13 HODE synthesized from LA and 5 oxo ETE and 5 HETE (Table 3).

## 4. Discussion

Erythrocytes are the most abundant blood cells, characterized by great flexibility that allows them to pass freely through the capillaries, transporting oxygen to the tissues and delivering carbon dioxide to the lungs [18]. The volume of erythrocytes and their state of hydration are regulated by the intracellular concentrations of monovalent cations, including low sodium (6–8 mM) and high potassium (102–130 mM). In plasma, sodium is present at a high level (140–150 mM) and potassium at a low level (4–5 mM) [19]. Potassium is an element that plays an important role in cellular metabolism and proper neuromuscular function. The distribution of potassium between the intracellular and extracellular environments is regulated by physiological factors, such as insulin and catecholamines, which stimulate the activity of Na^+^/K^+^ ATPase. Na^+^/K^+^ ATPase is known to be highly susceptible to oxidative stress [7,20]. Insulin carries potassium into cells by stimulating the activity of the Na^+^-H^+^ antiporter on the cell membrane, promoting the entry of sodium into the cells, which leads to the activation of Na^+^/K^+^ ATPase, causing an influx of potassium [5]. As is known, PCOS patients are characterized by disturbances not only in lipid metabolism but, above all, in carbohydrate metabolism, which leads to increased insulin levels. These are accompanied by an increase in free radicals and the development of oxidative stress in cells [21,22]. This may initiate disturbances related to the flow of potassium inside the cells. In addition, we found negative correlations between the level of potassium and the amounts of saturated fatty acids (pentadecanoic acid, palmitic acid and stearic acid) in the erythrocyte cell membrane. Increases in the amounts of these acids in cell membranes cause cell membrane stiffening and the deformation of erythrocytes [23]. This is probably another factor that contributes to the dysregulation of potassium transport into the cell interior. The relationship of saturated fatty acids in erythrocytes, and in particular palmitic acid, with total and cardiovascular mortality was recently demonstrated by Kleber et al. [24]. Saturated fatty acids (SFAs) in red blood cells, especially palmitic acid and stearic acid, have been associated with IL-6 and CRP, contributing to inflammation [25]. It, therefore, seems that due to the growth of factors and the action of destabilizing mechanisms, the transport of potassium to the cells among PCOS patients is reduced. In our study, however, we did not observe lower levels of potassium in the red blood cells of women with PCOS. However, it seems that the Na^+^/K^+^ pump is not able at some stage to compensate for the disturbances in the concentration of these electrolytes. Since modifying sodium, potassium, calcium, magnesium and trace element intake can affect arachidonic acid metabolism and eicosanoid production [26], perhaps we could elucidate the other mechanisms involved in the synthesis of pro-inflammatory mediators. The effect of AA on membrane currents, voltages and cytoplasmic Ca^2+^ concentrations has already been described, as has AA’s induction of highly conductive potassium channels and membrane hyperpolarization [27]. However, so far, no one has described the synthesis of pro-inflammatory AA mediators that may be involved in improving the function of potassium channels. A group of organic compounds, including the benzopyran series, includes powerful vasorelaxant drugs, such as cromakalim, an example of a potassium channel opener used in the treatment of cardiovascular diseases [28]. These drugs have broad therapeutic potential and can also be used more widely in the PCOS group.

Saturated fatty acids lengthen to synthesize arachidonic acid. This acid has, so far, not been sufficiently researched. It is known, however, that it is the main component of cerebrosides, i.e., the cells responsible for the construction of the myelin sheath of nerve fibres. Therefore, if the level of K decreases, the synthesis pathway of this acid with pro-inflammatory properties increases, and, reaching the arachidonic acid, is desaturated into unsaturated derivatives. Epidemiological studies indicate the role of potassium in the clinical manifestations of cardiovascular disease, linking low serum potassium with a significantly higher risk of cardiovascular mortality [29,30]. 

Despite the lack of available literature on the relationship of potassium with the synthesis pathway of proinflammatory mediators in PCOS, the results of our research indicate that the decreased potassium level in the erythrocyte membranes is associated with the synthesis of the HODE and 5LOX pathways.

Potassium channels play a key role in controlling the resting membrane potential and the excitability of sensory neurons. K^+^ channels are the most numerous and widespread class of ion channels in the neurons, governed by approximately 78 human genes [31]. The opening of these channels facilitates the hyperpolarized outflow of K^+^ through the cell membrane, which counteracts the internal conductivity of ions, reducing the excitability of neurons [32]. The voltage-gated potassium channels in myelinated neurons are covered with a thick myelin sheath. Fatty acids, including neuronic acid, are an important component of the myelin sheath. [33,34]. Damage to myelin has been shown to unmask potassium channels, creating abnormal potassium currents that inhibit conduction [35]. This may explain the positive correlation that exists between nervonic acid concentration and potassium.

In the cell membranes of sensory afferents, there are TRPV1 vanilloid receptors (transient receptor potential cation channel subfamily V member 1) [36]. The activation of these receptors leads to the regulation of blood flow and body temperature and is responsible for the release of insulin and cytokines [37]. The two products of the oxidation of linoleic acid, namely the acids 9 HODE and 13 HODE, have been shown to function as endovanilloids, i.e., endogenous agonists of the potential channels of the vanilloid-1 transient receptor potential (TRPV1) channels [37]. When TRPV1 is phosphorylated after ligand binding, the channel is opened, and cations (mainly Na^+^ and Ca^2+^) enter the intracellular space [38]. Potassium is responsible for increasing the permeability of cell membranes by antagonizing the action of calcium ions. It creates the resting and functional potential of nerve cells and takes part in the transmission of information by neurons. 

## 5. Conclusions

In our present state of knowledge, the effect of some fatty acids on the level of potassium in the erythrocyte membranes is difficult to analyse. However, it seems that SFA reduces the flow of potassium levels into the cell, destabilizing the pH of the cytosol, and thus exacerbating the inflammatory reaction that involves the AA acid cascade. The 9 and 13 HODE and 5 HETE synthesis pathways in the PCOS group are the most involved; therefore, improving the flow of potassium inside the cell is important in the treatment of patients.

## Figures and Tables

**Table 1:** The characteristics of the studied women with PCOS and the control group (CG).

Parameter	Women with POCS (n = 38)		Control Group (n = 17)		*p* Value *
	x ± SD	Range	x ± SD	Range	
Height (m)	1.68 ± 0.06	1.56–1.79	1.67 ± 0.06	1.56–1.77	0.7904
Body weight (kg)	80.7 ± 13.6	55.8–116.5	63.7 ± 9.1	52.4–80.0	0.0001
BMR (kcal)	1490 ± 119	1288–1963	1408 ± 162	1210–1785	0.0416
Na/K	0.97 ± 0.26	0.6–2.2	1.18 ± 0.56	0.80–3.0	0.0638
TBW (L)	35.9 ± 5.8	17–50.9	32.1 ± 6.3	15.3–43.4	0.0316
BMI (m^2^/kg)	29.0 ± 5.2	19.5–43.2	23.1 ± 3.5	18.4–31.9	0.0002
WHR (cm/cm)	0.92 ± 0.08	0.75–1.13	0.78 ± 0.07	0.75–1.0	0.0001
K (µg/mL)	2402 ± 247	2165–2600	2499 ± 364	2052–3447	0.2954

* Only the correlations that have *p*-values greater than 0.05 are marked in bold; BMR—basic metabolism rate; TBW—total body water; BMI—body mass index; WHR—waist–hip ratio.

**Table 2:** Correlations between the amount of potassium in red blood cells and fatty acids in women with PCOS.

Fatty Acids	r *	Fatty Acids	r *
C 8:0 caprylic acid	0.1661	C18:3n-6 gamma-linolenic acid	−0.2660
C10:0 capric acid	−0.1031	C18:3n-3 linolenic acid	−0.1235
C12:0 lauric acid	−0.2253	C20:0 arachidic acid	**−0.3612**
C14:0 myristic acid	−0.2702	C20:1 cis-11-eicosenoic acid	−0.1282
C14:1 myristoleic acid	0.0707	C20:2 cis-11-eicosadienoic acid	0.3087
C15:0 pentadecanoic acid	**−0.4219**	C20:3n6 cis-11-eicosatrienoic acid	**−0.5245**
C16:0 palmitic acid	**−0.4145**	C20:4n6 arachidonic acid	**−0.3933**
C16:1 palmitoleic acid	0.0202	C20:5n3 EPA	−0.1423
C17:0 heptadecanoic acid	−0.2609	C22:0 behenic acid	−0.0147
C17:1 cis-10-heptadecanoid acid	−0.0208	C23:0 tricosanoic acid	−0.1263
C18:0 stearic acid	**−0.5084**	C22:4n6 docosatetraenic acid	−0.3270
C18:1w9ct oleic acid	−0.0836	C22:5w3 docosapentaenoic acid	−0.1613
C18:1trans11 -vaccenic acid	−0.0657	C22:6w3 docosahexaenoic acid	−0.1236
C18:2n6c linoleic acid cis	−0.2702	C24:1 nervonic acid	**0.3857**

* significant correlations are marked in bold; r—correlation coefficient.

**Table 3:** Correlations between potassium levels and arachidonic acid (AA) derivatives.

AA Derivatives	r *	AA Derivatives	r *
LTX A4 5S, 6R, 15R	0.191	15S HETE	0.242
LTX A4 5S, 6R	−0.022	12S HETE	0.144
16RS HETE	−0.259	5 oxo ETE	**0.351**
13S HODE	**0.536**	5 HETE	**0.473**
9S HODE	**0.359**		

* significant correlations are marked in bold; r—correlation coefficient.

**Table 4:** Correlations of the anthropometric and biochemical parameters of PCOS patients in relation to the potassium levels in their red blood cells.

Parameter	PCOS Group (n = 38)		Correlations (r) between the Tested Parameter and the Level of Potassium *
	x ± SD	Range	
DHEA-SO_4_ (μg/d)	229.5 ± 85.6	61.5–392	0.023
Androstendione (ng/mL)	3.63 ± 1.56	1.2–8.2	−0.151
TSH (mIU/mL)	1.76 ± 0.60	0.81–4.02	0.279
LH (mIU/mL)	7.70 ± 3.45	3.85–18.3	−0.001
FSH (mIU/mL)	5.17 ± 1.20	2.70–8.81	−0.262
Estradiol (ng/mL)	46.27 ± 23.14	5.62–146.0	−0.198
Testosterone (ng/mL)	0.574 ± 0.238	0.23–1.30	0.186
Prolactin (ng/mL)	17.3 ± 6.6	2.9–34.5	0.182
Insulin (mU/L)	14.1 ± 10.3	3.5–64.4	0.372
Insulin after 2 h (mU/L)	88.4 ± 72.9	7.2–383.9	−0.060
Glucose (mg/dL)	92.6 ± 10.1	66.5–129.5	−0.174
Glucose after 2 h (mg/dL)	117.4 ± 30.1	79.2–219.3	−0.146
Cholesterol (mg/dL)	181.6 ± 26.4	122.6–242.0	0.039
LDL (mg/dL)	112 ± 26.9	58.7–181.0	−0.023
HDL (mg/dL)	57.1 ± 17.0	29–103.3	0.274
TG (mg/dL)	108.1 ± 51.3	40.8–264.8	−0.167

* No significant correlations. DHEA-SO4—dehydroepiandrosterone sulphate; TSH—thyroid-stimulating hormone; LH—luteinizing hormone; FSH—follicle-stimulating hormone; LDL—low-density lipoprotein; HDL—high-density lipoprotein; TG—triglyceride.

## Data Availability

The data presented in this study are available upon request from the corresponding author.

## References

[1] Youssef H.M.G., Marei E.S.M., Rashed L.A. (2019). Long non-coding RNA steroid receptor activator in polycystic ovary syndrome: Possible association with metabolic syndrome. Clin. Exp. Obstet. Gynecol..

[2] Neven A.C.H., Laven J., Teede H.J., Boyle J.A. (2018). A summary on polycystic ovary syndrome: Diagnostic criteria, prevalence, clinical manifestations, and management according to the latest international guidelines. Semin. Reprod. Med..

[3] Taher M.A., Mhaibes S.H. (2017). Assessment of Some Trace Elements in Obese and Non-Obese Polycystic Ovary Syndrome (PCOS). Int. J. Sci. Res..

[4] Palmer B.F., Clegg D.J. (2016). Physiology and pathophysiology of potassium homeostasis. Adv. Physiol. Educ..

[5] Li T., Vijayan A. (2014). Insulin for the treatment of hyperkalemia: A double-edged sword?. Clin. Kidney J..

[6] Sorensen M.V., Matos J.E., Praetorius H.A., Leipziger J. (2010). Colonic potassium handling. Pflug. Arch..

[7] Cetinkaya C.D., Gurbilek M., Koc M. (2018). Evaluation of the relationship of erythrocyte membrane Na+/K+-ATPase enzyme activity and tumor response to chemoradiotherapy in patients diagnosed with locally advanced nonsmall cell lung cancer and glioblastoma multiforme. J. Can. Res. Ther..

[8] De Rosa M.C., Alinovi C.C., Galtieri A., Scatena R., Giardina B. (2007). The plasma membrane of erythrocytes plays a fundamental role in the transport of oxygen, carbon dioxide and nitric oxide and in the maintenance of the reduced state of the heme iron. Gene.

[9] Gibka M., Grudzińska E., Fabian-Danielewska A. (2019). Analysis of changes in the morphology of erythrocytes—Microscopic evaluation of peripheral blood smears. J. Phys. Educ. Sport.

[10] Wang Y., Yang P., Yan Z., Liu Z., Ma Q., Zhang Z., Wang Y., Su Y. (2021). The relationship between erythrocytes and diabetes mellitus. J. Diabetes Res..

[11] Mohanty J.G., Nagababu E., Rifkind J.M. (2014). Red blood cell oxidative stress impairs oxygen delivery and induces red blood cell aging. Front. Physiol..

[12] D’Angelo S., Motti M.L., Meccariello R. (2020). ω-3 and ω-6 Polyunsaturated Fatty Acids, Obesity and Cancer. Nutrients.

[13] Djuricic I., Calder P.C. (2021). Beneficial Outcomes of Omega-6 and Omega-3 Polyunsaturated Fatty Acids on Human Health: An Update for 2021. Nutrients.

[14] Rotterdam ESHRE/ASRM-sponsored PCOS Consensus Workshop Group (2004). Revised 2003 consensus on diagnostic criteria and logterm health risks related to polycystic ovary syndrome (PCOS). Hum. Reprod..

[15] Norgan N. (2005). Laboratory and field measurements of body composition. Public Health Nutr..

[16] Raszeja-Wyszomirska J., Safranow K., Milkiewicz M., Milkiewicz P., Szynkowska A., Stachowska E. (2012). Lipidic last breath of life in patients with alcoholic liver disease. Prostaglandins Other Lipid Mediat..

[17] Szczuko M., Kotlęga D., Palma J., Zembroń-Łacny A., Tylutka A., Gołąb-Janowska M., Drozd A. (2020). Lipoxins, RevD1 and 9, 13 HODE as the most important derivatives after an early incident of ischemic stroke. Sci. Rep..

[18] Benedik P.S., Hamlin S.K. (2014). The physiologic role of erythrocytes in oxygen delivery and implications for blood storage. Crit. Care Nurs. Clin..

[19] Zimna A., Kaczmarska M., Szczesny-Malysiak E., Wajda A., Bulat K., Alcicek F.C., Zygmunt M., Sacha T., Marzec K.M. (2021). An insight into the stages of ion leakage during red blood cell storage. Int. J. Mol. Sci..

[20] Kamboj A., Kiran R., Sandhir R. (2006). N-acetylcysteine ameliorates carbofuran-induced alterations in lipid composition and activity of membrane bound enzymes. Mol. Cell Biochem..

[21] Mohammadi M. (2019). Oxidative Stress and Polycystic Ovary Syndrome: A Brief Review. Int. J. Prev. Med..

[22] Udensi U.K., Tchounwou P.B. (2017). Potassium Homeostasis, Oxidative Stress, and Human Disease. Int. J. Clin. Exp. Physiol..

[23] Spychalska J. (2012). Red blood cell membranopathies—Pathogenesis, clinical presentation and diagnosis. Hematologia.

[24] Kleber M.E., Delgado G.E., Dawczynski C., Lorkowski S., März W., von Schacky C. (2018). Saturated fatty acids and mortality in patients referred for coronary angiography—The Ludwigshafen Risk and Cardiovascular Health study. J. Clin. Lipidol..

[25] Mu L., Mukamal K.J., Naqvi A.Z. (2014). Erythrocyte saturated fatty acids and systemic inflammation in adults. Nutrition.

[26] Peplow P.V. (1992). Modification to dietary intake of sodium, potassium, calcium, magnesium and trace elements can influence arachidonic acid metabolism and eicosanoid production. Prostaglandins Leukot. Essent. Fat. Acids.

[27] Zheng H., Nam J.H., Nguen Y.H., Kang T.M., Kim T.J., Earm Y.E., Kim S.J. (2008). Arachidonic acid-induced activation of large-conductance potassium channels and membrane hyperpolarization in mouse B cells. Pflug. Arch. Eur. J. Physiol..

[28] Sebille S., De Tullio P., Boverie S., Antoine M.H., Lebrun P., Pirotte B. (2004). Recent developments in the chemistry of potassium channel activators: The cromakalim analogs. Curr. Med. Chem..

[29] Sun Y., Byon C.H., Yang Y., Bradley W.E., Dell’Italia L.J., Sanders P.W., Agarwal A., Wu H., Chen Y. (2017). Dietary potassium regulates vascular calcification and arterial stiffness. JCI Insight.

[30] Sahranavard T., Carbone F., Montecucco F., Xu S., Al Rasadi K., Jamialahmadi T., Sahebkar A. (2020). The role of potassium in atherosclerosis. Eur. J. Clin. Invest..

[31] Ocana M., Cendán C.M., Cobos E.J., Entrena J.M., Baeyens J.M. (2004). Potassium channels and pain: Present realities and future opportunities. Eur. J. Pharmacol..

[32] Tsantoulas C., McMahon S.B. (2014). Opening paths to novel analgesics: The role of potassium channels in chronic pain. Trends Neurosci..

[33] Rasband M.N., Trimmer J.S. (2001). Developmental clustering of ion channels at and near the node of Ranvier. Dev. Biol..

[34] Poliak S., Salomon D., Elhanany H., Sabanay H., Kiernan B., Pevny L., Stewart C.L., Xu X., Chiu S.Y., Shrager P. (2003). Juxtaparanodal clustering of Shaker-like K+ channels in myelinated axons depends on Caspr2 and TAG-1. J. Cell Biol..

[35] Shi R., Sun W. (2011). Potassium channel blockers as an effective treatment to restore impulse conduction in injured axons. Neurosci. Bull..

[36] De Petrocellis L., Schiano Moriello A., Imperatore R., Cristino L., Starowicz K., Di Marzo V. (2012). A re-evaluation of 9-HODE activity at TRPV1 channels in comparison with anandamide: Enantioselectivity and effects at other TRP channels and in sensory neurons. Br. J. Pharmacol..

[37] Premkumar L.S., Abooj M. (2013). TRP channels and analgesia. Life Sci..

[38] Zhai K., Liskova A., Kubatka P., Büsselberg D. (2020). Calcium Entry through TRPV1: A Potential Target for the Regulation of Proliferation and Apoptosis in Cancerous and Healthy Cells. Int. J. Mol. Sci..

